# Surgical treatment of isolated right ventricular metastasis from renal cell carcinoma

**DOI:** 10.1186/s40792-019-0733-3

**Published:** 2019-10-29

**Authors:** Shuhei Miura, Akira Yamada, Yutaka Iba, Ryushi Maruyama, Eiichiro Hatta, Yoshihiko Kurimoto

**Affiliations:** Department of Cardiovascular Surgery, Teine Keijinkai Hospital, 1-12 Maeda, Teine-ku, Sapporo, 006-8555 Japan

**Keywords:** Renal cell carcinoma, Right ventricle metastasis, Surgical tumor resection

## Abstract

**Background:**

Cardiac metastasis from renal cell carcinoma is an exceptional event, particularly when there is lack of inferior vena cava involvement. Only a few cases have been reported worldwide so far.

**Case presentation:**

We presented a case of a 58-year-old man diagnosed with isolated right ventricular metastasis of renal cell carcinoma in the absence of direct inferior vena cava extension, who underwent surgical tumor resection using cardiopulmonary bypass.

**Conclusions:**

Surgical resection of the cardiac mass with an understanding of the pathology is needed to prevent sudden death from acute heart failure or tumor embolism and improve the patient’s quality of life.

## Background

Renal cell carcinoma (RCC) represents 3% of all malignant tumors and approximately 30% of the patients diagnosed with RCC develop metastasis [1]. The most common metastatic sites include the lung, bones, soft tissues, liver, and central nervous system. While cardiac metastases from RCC are unusual, isolated right ventricular (RV) metastasis without vena cava involvement is exceedingly rare [2]. Therefore, discussion of multidisciplinary therapies and follow-up strategies for cardiac metastasis of RCC is essential to prevent the risk of sudden deaths. In this report, we present a case of surgical treatment of isolated RV metastasis from RCC in the absence of vena cava extension.

## Case presentation

A 58-year-old man was presented to our hospital with progressive dyspnea and atypical chest pain. Clinical examination found no signs of congestion and angina pectoris. At the age of 51 years old, he had undergone partial right nephrectomy as right RCC was detected. Two years post to nephrectomy, multiple lung metastases from RCC were detected, and the patient was treated with targeted molecular therapy (Sorafenib). This treatment happened to be successful with reduced and disappeared metastatic lesions. However, 4 years post nephrectomy, right adrenal gland metastasis was subsequently detected. He was treated with another targeted molecular therapy (Sunitinib), but limited effect was observed followed by increased lesion, and eventually underwent right adrenalectomy. Post these treatments, lung and adrenal gland metastases were well-controlled through chemotherapy.

**Fig. 1:**
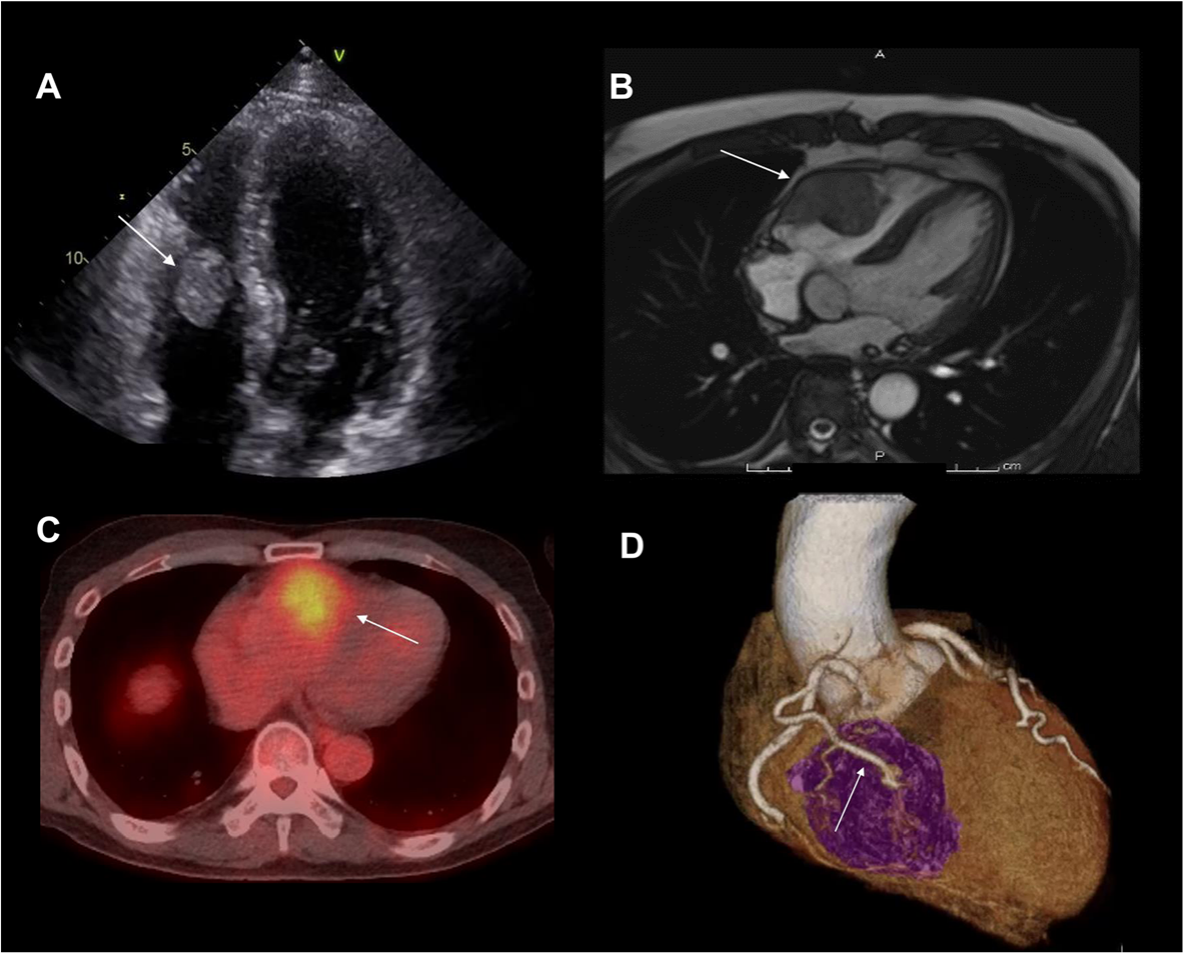
**a** Transthoracic echocardiography shows a 52 × 31 mm right ventricular mass (arrow) moving without extension into the outflow tract. **b** Cardiac MRI showed an anterior right ventricular free wall mass of the RV myocardium. **c** Axial fused FDG-PET/CT image demonstrates mildly FDG-avid mass within right ventricular wall (arrow). **d** A cardiac CT angiogram (segmented three-dimensional volume rendered image) shows the RV branches entering (arrow) the mass

At the time of admission, transthoracic echocardiography showed a 53 × 32 mm mass in the RV that moved without extension into the outflow tract nor involvement of the inferior vena cava (IVC) (Fig. 1a). Cardiac magnetic resonance imaging (MRI) confirmed a mobile mass with hypervascular tissue characteristics infiltrating the free wall of the RV myocardium (Fig. 1b). A fluorodeoxyglucose-position emission tomography (FDG-PET) examination showed a mildly FDG-avid mass in the RV free wall and free of other organ metastases (Fig. 1c). Contrast-enhanced cardiac computed tomography (CT) displayed an intramyocardial mass in the RV wall supplied by RV branch of the right coronary artery (Fig. 1d). After consultation with urology and oncology, the differential diagnosis included cardiac metastatic from RCC based on his medical history. Surgical tumor resection might enable preventing a tumor embolism-related sudden death as well as identifying appropriate anticancer agent through pathological diagnosis of metastatic lesion. Therefore, a multidisciplinary treatment was planned expecting a prognosis improvement.

**Fig. 2:**
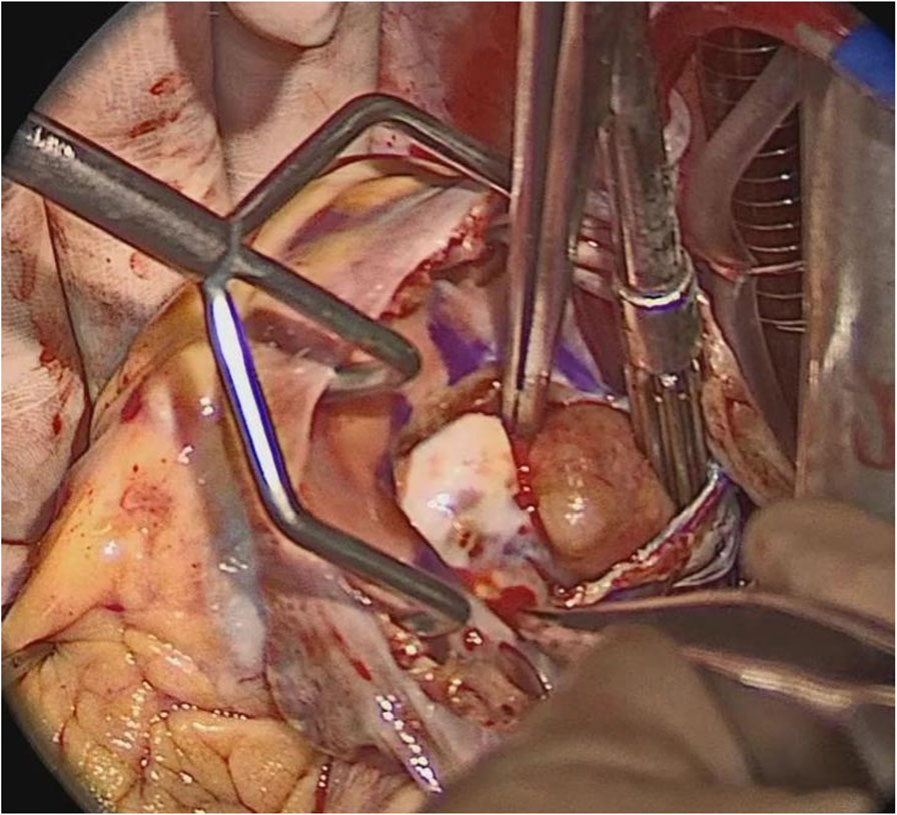
Intraoperative image. Giant tumor adhered to the free wall was deployed to the right atrium space by manual compression of the outside of RV wall via the tricuspid valve

**Fig. 3:**
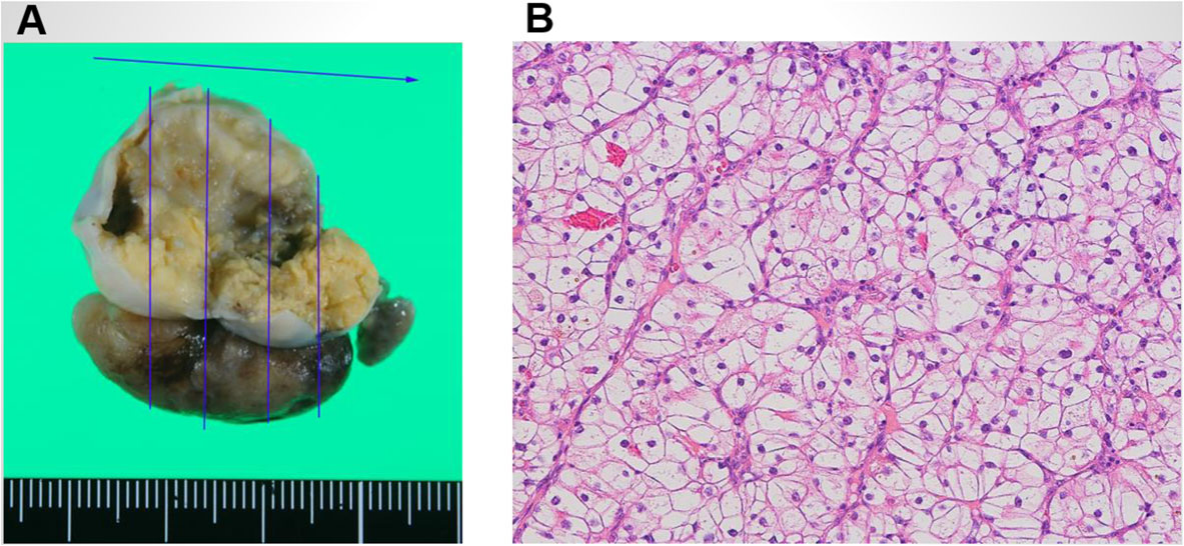
**a** Excised cardiac tumor after autopsy. **b** Microscopy image revealed a lot of proliferation of clear cytoplasm with abundant glycogen, which indicated the metastatic tumor from clear cell renal cell carcinoma (HE stains × 10)

Under general anesthesia and median sternotomy, a cardiopulmonary bypass was established between the ascending aorta and bicaval drainage. After cardiac arrest, the inside of the RV was observed with the approach via the tricuspid valve by the right atriotomy. A part of the valve leaflets between the anterior and posterior leaflet were incised near the annulus and the tumor was deployed to the right atrium by manual compression of the outside of the RV wall (Fig. 2). It was observed that the tumor layer attached to the RV free wall was thin, but the muscular layer remained as previous. Moreover, RV branch was running on the center of the lesion. For that reasons, the tumor was resected to preserve the RV wall as much as possible. Considering the possibility of tumor cell remnants, cryoablation was managed against the wall followed by a tricuspid annuloplasty with a 26-mm Physio tricuspid (Carpentier-Edwards, Irvine, California) after the leaflet suture repair. The operation progressed straightforward and weaning from the cardiopulmonary bypass was smooth. Histopathological examination led to a final diagnosis of metastatic tumor from clear cell RCC (Fig. 3). Postoperative echocardiography showed the disappearance of RV tumor and normal tricuspid valve function. The patient was discharged on postoperative day 16 after an uneventful hospitalization. One year post surgery, he has been asymptomatic and stable course without recurrence of cardiac tumors and metastasis to other organs under careful follow-up of oncologists.

## Discussion

Approximately 45% of the patients with RCC present localized tumors, 25% of patients present locally advanced disease, and approximately 30% of patients may have metastases at the time of diagnosis [1]. Cardiac metastases of RCC occur through two mechanisms. The first is a lymphatic pathway through the lymphatic vessels of the thorax collecting the drainage from the posterior wall of the heart. Reports have mentioned that drainage from the left heart wall passes through these lymph vessels and lymphatic flow can be reversed by metastasis to the nodes [3]. The second mechanism involves a venous hematogenous pathway through the renal vein to the right heart. In cases with isolated and delayed progression to the right heart without involvement of the IVC remain the most probable mode of metastasis through venous hematogenous micro dissemination [2]. This mechanism of metastasis is more compatible with the present case because of an isolated lesion and the right heart.

Patients with cardiac metastases present nonspecific symptoms such as palpitations, chest pain, shortness of breath, and syncope. Coronary occlusion or compression from tumor masses can lead to myocardial infarction, eventual heart failure, and even death [4]. A high index of suspicion is required to make a timely diagnosis of cardiac metastases because of the nonspecific clinical symptoms. Although various diagnostic imaging modalities have been used in prior reports, cardiac MRI is recommended as a reliable tool for evaluating the cardiac masses given its excellent contrast resolution and tissue characterization, which can exclude lipomas, fibromas, and hemangiomas as well as thrombus or lipomatous hypertrophy [5]. Also, cardiac CT provides high-quality images with superior spatial resolution for evaluation of relationship between the tumor and coronary arteries for surgical planning for the mass resection [6].

Unlike most other neoplasms, metastatic RCC is relatively resistant to conventional chemotherapy. Moreover, angiogenesis inhibitors, cytokine-based therapy including interferon, were the mainstay of treatment for advanced RCC. The development of drugs known as receptor tyrosine kinase inhibitors including sorafenib and sunitinib has created a paradigm shift in the treatment of RCC. Some reports have described cases of sudden death due to malignant cardiac metastases [7]. However, there is no consensus regarding surgical treatment for such disease. While the patients with isolated cardiac metastasis of RCC generally have obstructive symptoms; surgical resection may provide effective and favorable outcomes by preventing tumor embolism [8]. In this case, the free wall of the tumor adhesion site was thinned and the muscular layer remained. Moreover, RV branch was running on the center of the tumor lesion. Therefore, the transmural wall resection was not performed to avoid postoperative RV dysfunction by over-invasive surgery. In addition, cryoablation was managed against the RV wall to prevent tumor cell remnants. A pen-type freeze coagulation device frequently used in maze procedure for atrial fibrillation was adopted. The procedure was adopted after referring to some multidisciplinary treatment combining hepatectomy, microwave coagulo-necrotic therapy (MCN), and postoperative chemotherapy. It has been reported that this treatment may provide long-term survival for patients with unresectable metastatic hepatocellular carcinoma [9, 10]. However, there is no report on the effectiveness of MCN for metastasis of renal cell carcinoma. Surgical resection acts as palliation therapy of malignant cardiac metastasis; thus, multidisciplinary therapy as a combination of surgical treatment and targeted molecular therapy with cooperation of multiple experts is essential. For carefully selected patients, surgical resection of cardiac metastases to provide symptom palliation, improved quality of life, and prolonged survival may be acceptable.

## Conclusions

Here, we report a surgical tumor resection in a patient with RV mass caused by metastatic RCC. Since there is a risk of tumor recurrence affected by the tumor cell remnants or ineffective chemotherapy, close observation should be mandatory. Our experiences highlight the necessity of excision of the cardiac mass surveillance to improve quality of life.

## Data Availability

All related data are included within the article.

## Abbreviations

CT: Computed tomography
FDG-PET: Fluorodeoxyglucose-position emission tomography
IVC: Inferior vena cava
MRI: Magnetic resonance imaging
RCC: Renal cell carcinoma
RV: Right ventricular
